# The Relationship between Music and Food Intake: A Systematic Review and Meta-Analysis

**DOI:** 10.3390/nu13082571

**Published:** 2021-07-27

**Authors:** Tianxiang Cui, Jiaxuan Xi, Chanyuan Tang, Jianwen Song, Jinbo He, Anna Brytek-Matera

**Affiliations:** 1 School of Humanities and Social Science, The Chinese University of Hong Kong (Shenzhen), Shenzhen 518172, China; tianxiangcui@link.cuhk.edu.cn (T.C.); jiaxuanxi@link.cuhk.edu.cn (J.X.); chanyuantang@link.cuhk.edu.cn (C.T.); jianwensong@link.cuhk.edu.cn (J.S.); 2 Institute of Psychology, University of Wroclaw, 50-527 Wroclaw, Poland

**Keywords:** music, food intake, body mass index, meta-analysis, systematic review

## Abstract

Food intake has been shown to be related to several environmental factors including the presence of music. However, previous findings of the relationship between music and food intake are inconsistent. In the present study, a systematic review and meta-analysis was conducted to quantitatively review the extent to which music is associated with food intake as well as to investigate potential moderators that might have contributed to the heterogeneity of the existing findings. Literature was searched on four databases (i.e., PsycINFO, Web of Science, PubMed, and ProQuest Dissertations and Theses) and Google Scholar. Nine articles published from 1989 to 2020 met our inclusion criteria. A meta-analysis was carried out via a three-level random-effects model. The overall effect size (i.e., Hedges’ g) was 0.19 (95% Confidence Interval: −0.003, 0.386; *SE* = 0.10, *t* = 1.99, *p* = 0.054), indicating a marginally significant but small effect size. Body Mass Index (*F*(1, 21) = 5.11, *p* = 0.035) was found to significantly contribute to the heterogeneity of effect sizes, with larger positive effects of music on food intake for individuals with higher BMI. However, music-related features did not significantly moderate the relationship between music and food intake. More experimental studies are needed to update the current meta-analysis and get a better understanding of this topic.

## 1. Introduction

Food intake is about how much food an individual consumes. Food intake can be influenced by internal psychological factors and external factors. Internal factors include hormones [1], blood glucose [2], and genes [3,4]; while external factors include both environmental and social aspects [5,6,7,8,9]. Environmental factors can be divided into those associated with an eating environment and those associated with a food environment [9]. The former includes ambient factors that are not directly related to food (e.g., atmospherics, the effort of obtaining food, social interactions, and eating distractions), while the latter involves factors that are directly related to food (e.g., its salience, structure, and size). When people are exposed to environmental factors such as the salience of food (e.g., the food is in an eye-catching position), social aspects of eating (eating with others), and/or eating distractions (eating when watching TV or reading a book), food intake can be affected [9,10].

Music is a common environmental stimulus that can be present in both public and private eating places. It has been demonstrated that music affects food intake [8,11,12], and specifically in relation to the various features of music [13]. Musical features can include genre, tempo, volume, the presence or absence of an accompanying human voice, and familiarity with music [14,15]. In particular, loud music [16], music with vocals [17,18], and familiar music [19,20] have been shown to contribute to greater arousal by changing psychological responses (e.g., heart rate, blood pressure) than music with opposite characteristics (e.g., soft music, music without vocals, and unfamiliar music) [12]. With greater arousal, an individual can become more distracted and disregard a feeling of fullness while eating [21,22]. This potentially represents how music can affect food intake [12,23,24] and a similar mechanism was found where mealtime stimuli were affected by television viewing and listening to recorded stories [23]. The relationship between music and food intake was also explored from the emotional perspective. Previous experimental studies found that music valence (musical positiveness conveyed by a music track) influenced food preferences [25,26], thereby affected food intake. This phenomenon was defined as the sensation transference effect [27]. Similarly, Kantono et al. [28] regarded emotion as a mediator between music valence and food perception. Besides, emotions were thought to affect food intake in multiple ways [29].

Conversely, however, there are other studies that have not found that music influences food intake [30,31,32]. To the best of our knowledge, no systematic reviews and/or meta-analyses have explored the relationship between music and food intake. Therefore, in order to clarify the inconsistent findings published regarding the influence of music on food intake, we conducted a systematic review to investigate potential moderators by using a meta-analytic approach.

### 1.1. Literature Review

#### 1.1.1. Volume of Music

The relationship between the volume of music and food intake has previously been investigated. While some studies found that louder music is related to higher consumption of beverages and alcohol [33,34,35] where emotion was considered as a mediator [27], other studies have found no effect of the volume of music on food intake. However, it has been observed that the presence of music does increase food consumption [12]. Therefore, further investigation is needed to determine whether the volume of music moderates the relationship between music and food intake.

#### 1.1.2. Type of Music

It has also been demonstrated that different types of music distinctly impact the relationship between music and food intake. For example, rock music has correlated with increased consumption of alcohol [36], while classical music appears to help reduce the intake of savory foods [37]. People also tend to spend more money at a restaurant when classical or jazz music is playing rather than pop music or no music at all [38]. Thus, to some extent, the spending of money is related to the amount of food consumed. Food enjoyment is also positively related to food intake [39], while classical music can increase food enjoyment as well [40]. Thus, it is relevant to explore whether types of music moderate the relationship between music and food intake.

#### 1.1.3. Speed of Music

In the presence of high tempo songs, a higher intake of foods has been observed [41,42,43]. Tempo has also been related to the speed of food intake, which is relevant to the volume of food consumed [44]. For example, people are likely to drink faster when listening to fast-tempo music [45], and chewing intensity, namely, the number of bites, can also increase with higher tempo music [42]. Conversely, when people are exposed to music with a slower tempo, they tend to eat more slowly and pay more in restaurants [41,46]. However, music tempo has not been found to significantly affect food intake [12]. Hence, it is critical to explore whether the speed of music moderates the relationship between music and food intake.

#### 1.1.4. Variety of Food Type

A greater variety of food types has the potential to increase food intake and may represent a confounding variable of the energy density of foods [11]. It has been observed that a variety of food types did not increase food intake when different types of vegetables (a low energy density food) were offered [47,48].

#### 1.1.5. Eating Location and Sample Size

According to a previous study [12], music may have a more significant effect on food intake when a larger sample size is presented; therefore, it is possible that the effects of music appear differently in different eating locations (e.g., in a restaurant, at home, in a car).

## 2. Materials and Methods

### 2.1. Literature Search

A literature search was conducted to identify studies published prior to December 2020 which explored the relationship between music and food intake. To confirm databases, keywords for a literature search, as well as selection criteria for filtering eligible studies, and previous meta-analyses with relevant topics were referred [49,50,51,52,53,54,55,56]. There were four relevant databases identified, PsycINFO, Web of Science, PubMed, and ProQuest Dissertations and Theses (PQDT), and these were used to conduct our literature search. We also used Google Scholar as a supplementary database for the literature search. Keywords that were used included both the extended words of music and food intake, namely (music OR musical experience OR musical preference OR musical skills OR musical abilities OR auditory discrimination OR music interventions OR effects of music OR musicians OR lullaby OR sing OR song OR listening OR harp OR auditory stimulation) AND (food intake OR eating OR food consumption OR ingestion OR food choice OR diet OR appetite OR satiety OR satiation OR “expected satiety” OR appetite OR hunger OR fullness OR “sensory-specific satiety” OR “energy intake” OR “food behavior” OR “eating behavior”).

### 2.2. Selection Criteria

Articles were selected according to the following inclusion criteria: (a) published in English; (b) the amount of food consumed by participants/energy intake was measured (evaluation of food choice, self-reported food intake, behavioral intentions, or other measures did not qualify); (c) surveys and experiments were both included, and; (d) there was no age limitation for participants. Conversely, articles were excluded if: (a) their abstracts did not include sufficient information to code; (b) they represented theoretical review papers; (c) they duplicated data from another study, or; (d) they employed two or more approaches (e.g., music plus videos). Comparisons between music-on versus music-off conditions while presented with music-related stimuli were (e.g., television viewing) were also excluded. Thus, only music-off conditions without music-related stimuli were included (e.g., eating alone, eating in groups, etc.).

It has been observed that systematic review-based meta-analyses with language restrictions for searching literature are less likely to lead to systematic bias [57]. Therefore, only articles written in English were selected, which is a common criterion for meta-analyses [58]. Dissertations were the only unpublished sources included in our literature search. There were several reasons for this. First, overestimating the treatment effect is rare when unpublished articles are excluded [59]. Secondly, dissertations are easier to access than other types of unpublished literature, and also tend to have low bias and complete demonstration of findings [60]. Third, dissertations are preferable to nonindexed unpublished studies to represent missing data [61].

### 2.3. Coding of Studies

Two authors (T.C. and J.X.) independently coded all the studies examined. Initially, the two authors learned to code from several articles [12,37,62,63]. In addition to the potential moderators mentioned in the previous section, other potential moderators were identified and confirmed via discussion with J.H. After gaining familiarity with the coding process, formal coding was initiated. Final intercoder reliability ranged from 0.90 to 1.00 (Krippendorff’s alpha). Discrepancies between the two coders were resolved after discussions with J.H.

The following information was extracted from the studies selected for this study: (1) name of the author; (2) publication year; (3) type of publication (journal or dissertation); (4) country (America or Europe); (5) experimental design (between group, within group or mixed design); (6) type of music (pop, classical, rock or other); (7) volume of music (high volume: higher or equals 68dB; low volume: lower than 68dB); (8) source of sample (high school, university, clinical context or other source); (9) meal duration (autonomous or controlled); (10) type of food (solid, liquid or mixed); (11) experimental setting (laboratory, natural environment or other settings); (12) number of participants; (13) number and proportion of male participants; (14) proportion of white participants; (15) mean of age and body mass index (BMI) of participants, and; (16) information for calculating standardized mean difference effect sizes (e.g., number of participants, mean (M), and standard deviation (SD) of food intake for two groups). There are two widely used standardized mean difference effect sizes, namely, Cohen’s *d* and Hedge’s *g*. Since Cohen’s *d* is likely to bias the estimate of effect size in the presence of small samples, we used Hedge’s *g* instead, which represents an unbiased version of Cohen’s *d* [64]. In the current meta-analysis, a positive value for *g* represented higher food intake in music-on groups, while a negative value for *g* represented higher food intake in music-off groups. To interpret effect sizes of *g*, cutoff values of 0.20, 0.50, and 0.80 were considered small, medium, and large, respectively [65].

### 2.4. Quality Assessment

Quality assessment for all the included studies was independently conducted by two authors (T.C. and J.X.). The tool for quality assessment was adapted from the scale used in a previous review [66] for assessing the quality of studies that investigated the relationship between music and food intake. The tool contains six items (sample item: “Did the study describe the participant eligibility criteria?”) which assess six aspects of study quality (e.g., participant eligibility criteria, sampling method, features of music, features of food (intake), power calculation, and critical statistics). Each response scale ranges from 1–6 (1–2 = low; 3–4 = medium; 5–6 = high). A methodological appraisal score is obtained by dividing the total score obtained by 36 (the total possible score), then multiplying it by 100. Percentage ranges of 0–33%, 34–66%, and 67–100% suggest poor, adequate, and good study quality, respectively.

### 2.5. Data Analyses

All data analyses were conducted with R 4.0.0 software [67] and the *metafor* package [68]. Traditional meta-analytic methods assume independent effect sizes; that is, effect sizes extracted for a meta-analysis are assumed to be independent. However, when multiple effect sizes are from a single study, the effect sizes are inevitably dependent on each other. The three-level meta-analysis [69,70,71] was developed to account for the dependency of effect sizes (e.g., multiple effects sizes from the same study). In the three-level meta-analysis, heterogeneity is from three levels of variance [71]: sampling variance of effect sizes (Level 1 variance), variance between effect sizes of the same sample (Level 2 variance), and variance between studies included in the analysis (Level 3 variance). Given that we extracted multiple effect sizes in almost all studies included; thus, we used a three-level random-effects model for our subsequent analyses.

Outlier detection is a critical step in a meta-analysis since outliers may influence the results (e.g., validity and robustness) [68]. In the present study, a Baujat plot and influential case diagnostics were employed to detect outliers [68,72]. Once detected, outliers were excluded from further data analysis steps to increase the precision of the estimated mean effect size from the random-effects model [68]. Thus, the overall effect size was calculated after outliers were removed.

*Q* statistics were used to determine if significant heterogeneity exists among the effect sizes. If heterogeneity among the effect sizes is significant, moderator analyses were conducted to examine what characteristics coded from the included studies might have contributed to this heterogeneity. In accordance with previous literature [58], if only a limited number of effect sizes exist for the categorical moderator variables (e.g., one or two studies), the subgroup was not included in the moderator analyses. For the continuous moderator variables, those containing less than ten effect sizes were not considered in the moderator analyses [73].

To evaluate whether publication bias was present, a funnel plot was generated and its symmetry examined with Begg’s rank test [74]. A non-significant *p*-value (e.g., >0.05) indicates insufficient publication bias.

## 3. Results

### 3.1. Study Selection

**Figure 1:**
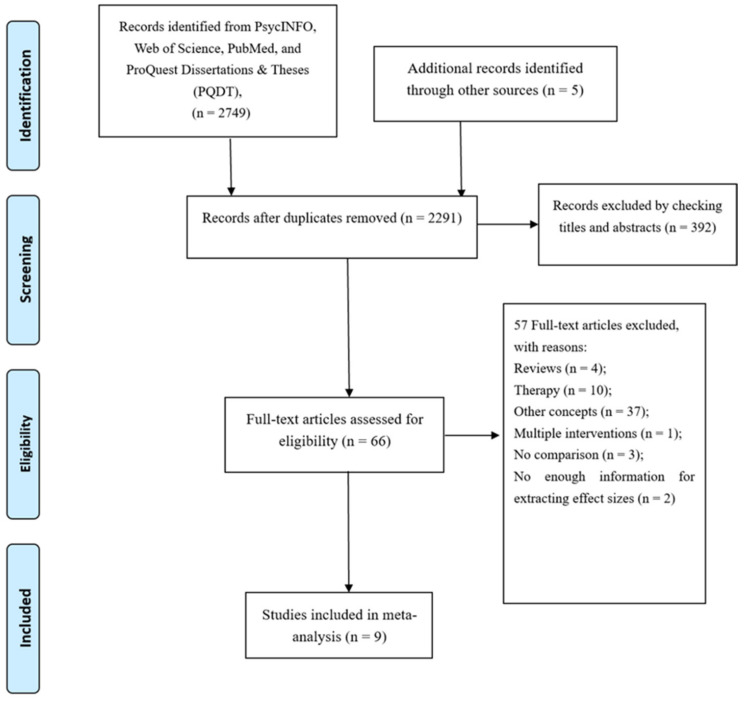
Flow diagram of the search strategy and study selection in this study.

The procedure used to select data is described in Figure 1. Briefly, the initial search identified 2754 records from four databases and Google Scholar. After removing duplicates, 2291 studies remained. A check of titles and abstracts identified 66 articles for full-text review. A total of nine articles provided sufficient information to be included in our meta-analysis.

### 3.2. Descriptive Characteristics of the Included Studies

The nine articles selected were published between 1989 and 2020 and included a total of 631 participants (252 males, 379 females). Forty-one effect sizes (Hedge’s g) were extracted from the articles (see Supplementary Materials, Table S1), and effect sizes ranged from −0.78 [37] to 3.15 [34]. Thus, large variability in the findings from these studies was observed.

### 3.3. Quality Assessment of the Included Studies

Quality appraisal scores for the identified primary studies ranged from 50.0% to 100.0% (see Supplementary Materials, Table S2), thereby reflecting an overall adequate quality of the included studies. Consequently, none of the studies were excluded because of poor quality.

### 3.4. Outlier Detection

Three effect sizes (No. 24 [34], No. 25, and No. 26 [75]; see Supplementary Materials, Figure S1) were detected as outliers according to the Baujat plots generated and influential case diagnostics. Outliers in a meta-analysis may bias the pooled effect size and can also affect the validity and robustness of the conclusions from a meta-analysis [72]. Therefore, these three outliers were excluded, resulting in 38 effect sizes included in subsequent analyses.

### 3.5. Overall Analysis

**Figure 2:**
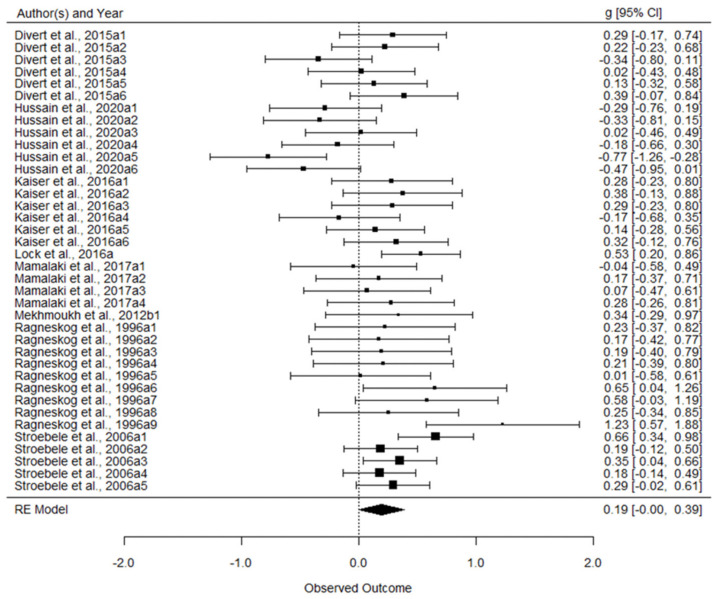
Forest plot of effect sizes (g) synthesis.

After excluding the identified outliers, the 38 remaining effect sizes were found to range from −0.78 [37] to 2.12 [75]. Using a three-level meta-analysis approach, the pooled effect size was found to be marginally significant, *g* = 0.191 (95% confidence interval (CI): −0.003, 0.386; standard error = 0.10, *t* = 1.99, *p* = 0.054). A forest plot of effect sizes synthesis is shown in Figure 2. Statistically significant heterogeneity, *Q_(37)_* = 71.46 (*p* < 0.001) was observed among these effect sizes. Therefore, moderator analyses were further conducted to explore which factors may have contributed to this heterogeneity.

### 3.6. Publication Bias

**Figure 3:**
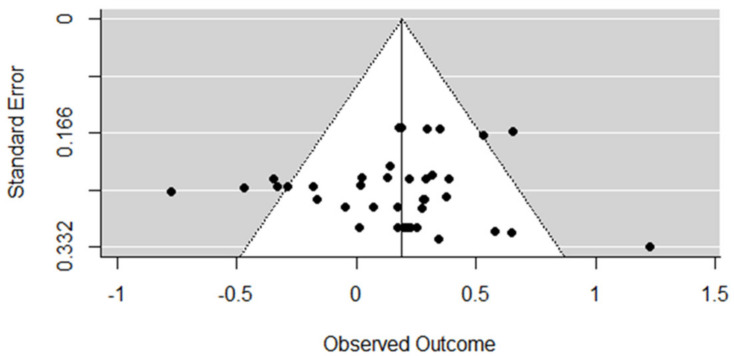
Funnel plot for effect sizes (g) synthesis.

A rank correlation test for funnel plot asymmetry was applied to all the included studies, and no evidence for publication bias was observed (Kendall’s tau = 0.07, *p* = 0.530) (Figure 3). Thus, publication bias is less likely to affect the pooled results of the present study.

### 3.7. Moderator Analyses

Among the proposed moderators, only the moderator effect of body mass index (BMI) was statistically significant (*F*_(1, 21)_ = 5.11, *p* = 0.035; *Q*_(21)_ = 26.63, *p* = 0.183) (Table 1). In addition, the pooled effect size of the music-on group was significantly larger than that of the music-off group (*β*_1_ = 0.114, *p* = 0.009). Detailed results for the other moderators with nonsignificant effects are presented in Table 1.

## 4. Discussion

We performed a systematic review and meta-analysis of the literature published up to December 2020 regarding the relationship between music and food intake. For the nine studies selected, we examined the overall association between music and food intake and the moderators which could potentially account for the heterogeneity observed among the studies we selected. We used a meta-analysis approach based on a three-level random-effects model. In the meta-analysis, music was found to be positively related to food intake, with a small effect, which is generally consistent with previous findings (e.g., [12,63]). BMI was the only significant moderator identified contributing to the heterogeneity across previous studies, suggesting that individuals with a higher BMI are more likely to have a greater intake of food and/or drink when music is present. To our knowledge, this conclusion has not been reported before. We assumed that individuals with a higher BMI may be more sensitive to food-related stimuli and consume a greater volume of food under stimuli than people with a lower BMI. However, the mechanism of how BMI may alter the relationship between music and food intake requires further experimental investigations.

Our observations regarding other potential moderators which were not statistically significant are consistent with the results of previous studies. For example, neither volume nor speed of music appeared to have an effect on food intake, consistent with the results of a prior study [12]. Previous studies which have reported significant effects of volume and speed of music on food intake were mainly conducted in a restaurant environment [33,41,42,43]. In contrast, the studies included in our meta-analysis were conducted primarily in private locations or laboratories (see [11,12,37]). It is possible that different eating locations may result in contradictory conclusions in previous studies. Furthermore, previous studies conducted in restaurants generally did not report sufficient information to perform a meta-analysis. Thus, most of them were excluded from our study. Considering that statistical power is an important issue in moderator analysis of a meta-analysis and the number of studies is one of the main determinants of statistical power [76], the lack of enough number studies may account for the nonsignificant findings of the moderator, experimental setting, in our meta-analysis.

When coding data from studies, there are some characteristics that may need additional specifics. It is also widely recognized that the classification of music types is complex. To simplify the coding results for the moderator analysis in this study, we coded only four types of music: pop, classical, rock, and other. However, if types of music are coded too precisely, there are some genres that are too rare to be representative types. Moreover, if we roughly coded the types, there are some important types of music (e.g., jazz, rap) that may be overlooked in a moderator analysis. Tempo or speed of the music appeared to be an important potential moderator in our study, yet the details of tempo were not clearly reported in many previous studies (e.g., [76]). Therefore, we did not code tempo in our analysis even though we tried to extract tempo-related information from the studies we selected. After some discussion, meal duration was also identified as a potential moderator. We classified meal duration as autonomous (e.g., participants were allowed to consume food without a time limitation) or controlled (e.g., researchers regulated the amount of time given for food consumption). Previously, autonomous meal duration has been found to be affected by the presence of music [12]. However, it remains unclear whether autonomous meal duration should be considered a potential moderator. Thus, meal duration is another moderator to further consider and investigate in future research.

There are limitations associated with the present study. A major limitation is that a limited number of articles (*n* = 9) were included for the meta-analysis, which may make the overall analysis and moderator analyses lack enough statistical power to detect the effects. Additionally, due to the small number of studies included, readers should be cautious to generalize the findings from the overall analysis and moderator analyses in the current meta-analysis. Moreover, previous studies mainly focused on eating behaviors in public areas such as restaurants (e.g., [34]), while the effect of music on food intake was not sufficiently explored through randomized controlled trials. Therefore, it is highly recommended that more experimental studies be conducted on the relationship between music and food intake to gain a better understanding of the topic.

## 5. Conclusions

Overall, our study summarized the findings from literature published up to December 2020 to explore the relationship between music and food intake. Results suggested that music is positively related to food intake with a small effect size. BMI was the only moderator identified that contributed to the heterogeneity across previous studies, suggesting that individuals with a higher BMI are more likely to have a greater intake of food and/or drink in the presence of music. Music-related features (volume, tempo, type, etc.) did not show a moderating effect between music and food intake. To confirm the present findings, more experimental studies are needed to further investigate the relationship observed between music and food intake (especially in relation to dietary intake) and to gain a better understanding of underlying moderators.

## Figures and Tables

**Table 1:** Results of Moderator Analyses.

Moderator Variables	No. Studies	No. ES	β_0_	95% CI	β_1_	95% CI	*F (df1, df2)*	Level 2 Variance	Level 3 Variance
Publication year	8	38	36.827	(−6.978; 80.631)	−0.018	(−0.040; 0.004)	2.878 (1, 36)	0.005	0.037 *
Percentage males	8	38	0.137	(−0.261; 0.535)	0.121	(−0.619; 0.861)	0.109 (1, 36)	0.004	0.064 ***
Age (year)	8	38	0.144	(−0.233; 0.521)	0.001	(−0.007; 0.009)	0.100 (1, 36)	0.004	0.066 ***
BMI	6	23	−2.574 *	(−5.097; −0.50)	0.114*	(0.009; 0.220)	5.110 (1, 21) *	0.002	0.028
Country									
United States	1	5	0.330	(−0.201; 0.861)	–	–	0.323 (1, 36)	0.004	0.063 ***
Europe	7	33	0.168	(−0.054; 0.391)	0.161	(−0.415; 0.738)			
Experimental design									
Between group	3	8	0.105	(−0.277; 0.488)	–	–	0.263 (2, 35)	0.005	0.068 *
Within group	3	18	0.280	(−0.052; 0.613)	0.175	(−0.332; 0.682)			
Mixed	2	12	0.162	(−0.240; 0.565)	0.057	(−0.498; 0.613)			
Type of music									
Pop	4	9	0.217	(−0.044; 0.479)	–	–	0.471 (2, 35)	0.007	0.045
Classical	1	3	0.025	(−0.404; 0.455)	−0.192	(−0.594; 0.210)			
Other	6	26	0.194	(−0.008; 0.396)	−0.023	(−0.294; 0.247)			
Volume of music									
High	3	5	0.341	(−0.655; 1.337)	–	–	0.277 (1, 16)	0.004	0.114 **
Low	4	14	0.072	(−0.358; 0.502)	−0.269	(−1.354; 0.816)			
Source of sample									
High school	1	1	0.341	(−0.534; 1.216)	–	–	0.311 (3, 34)	0.004	0.079 ***
University	3	17	0.073	(−0.275; 0.422)	−0.268	(−1.210; 0.674)			
Clinical context	2	15	0.245	(−0.185; 0.675)	−0.096	(−1.071; 0.879)			
Other	2	5	0.311	(−0.154; 0.777)	−0.030	(−1.021; 0.962)			
Meal duration									
Autonomous	6	33	0.193	(−0.037; 0.423)	–	–	0.000 (1, 36)	0.004	0.065 ***
Controlled	2	5	0.192	(−0.290; 0.674)	−0.001	(−0.536; 0.533)			
Type of food									
Solid	5	20	0.046	(−0.226; 0.318)	–	–	0.958 (1, 28)	0.000	0.060 **
Mixed	4	10	0.202	(−0.107; 0.512)	0.156	(−0.171; 0.484)			
Experimental setting									
Lab	4	17	0.033	(−0.239; 0.305)	–	–	1.292 (2, 35)	0.004	0.048 **
Natural	3	12	0.305 *	(0.014; 0.596)	0.272	(−0.127; 0.671)			
Other	1	9	0.373	(−0.119; 0.865)	0.340	(−0.222; 0.903)			
Experiment duration					–	–			
Long	4	22	0.246	(−0.051; 0.543)	–	–	0.257 (1, 36)	0.004	0.063 *
Short	4	16	0.143	(−0.142; 0.428)	−0.103	(−0.514; 0.309)			

Note. CI = Confidence interval; BMI = Body Mass index; Level 2 variance = Variance in effect sizes within studies; Level 3 variance = Variance in effect sizes between studies; * *p* < 0.05, ** *p* < 0.01, *** *p* < 0.001.

## Data Availability

The dataset used during the current study are available from the corresponding author on reasonable request.

## Supplementary Materials

The following are available online at https://www.mdpi.com/article/10.3390/nu13082571/s1, Figure S1: Outlier Detection—Baujat Plot, Table S1: Study Characteristics, Table S2: Quality Assessment of the Studies Examined.
