# Supplementary material for: The Relationship between Music and Food Intake: A Systematic Review and Meta-Analysis
Source: Nutrients. 2021 Jul 27;13(8):2571. doi: 10.3390/nu13082571 (PMC8400481; doi:10.3390/nu13082571)
Supplement: Supplementary file 1 [file nutrients-13-02571-s001.zip › nutrients-1298537-supplementary.pdf]

Online Supplementary Material

The Effect of Music on Food Intake: A Quantitative Systematic Review with Meta-Analysis

Tianxiang Cui[1], Jiaxuan Xi[1], Chanyuan Tang[1], Jianwen Song[1], Jinbo He[1, *] Anna Brytek-Matera[2, *]

[1] School of Humanities and Social Science, The Chinese University of Hong Kong (Shenzhen), Shenzhen, China.

[2] Institute of Psychology, University of Wroclaw, Wroclaw, Poland

* Corresponding authors:

Jinbo He, School of Humanities and Social Science, The Chinese University of Hong Kong, Shenzhen, 518172, China. Email: anlfhe@gmail.com; hejinbo@cuhk.edu.cn.

Anna Brytek-Matera, Institute of Psychology, University of Wroclaw, Wroclaw, 50-527, Poland. Email: anna.brytek-matera@uwr.edu.pl.

Supplementary Figure S1

Outlier Detection - Baujat Plot

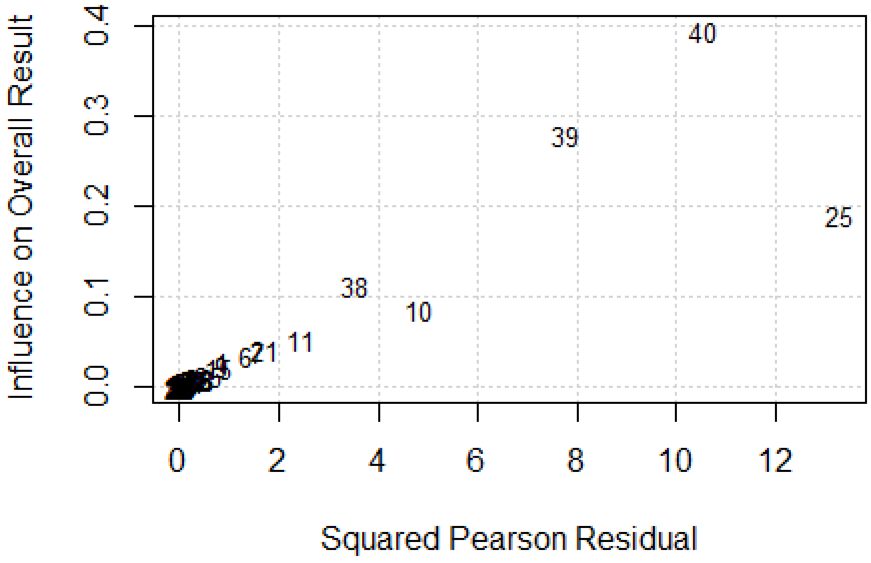

Supplementary Table S1

Study Characteristics

Author (s) and year	Publication type	Location of study	Sample size	No. of males	% males	Mean age (y)	Mean BMI (kg/m^2)	Experimental design	Music type
Divert et al., 2015a1	1	2	42	13	30.95	86.6	N/A	3	4
Divert et al., 2015a2	1	2	42	13	30.95	86.6	N/A	3	4
Divert et al., 2015a3	1	2	42	13	30.95	86.6	N/A	3	4
Divert et al., 2015a4	1	2	42	13	30.95	86.6	N/A	3	4
Divert et al., 2015a5	1	2	42	13	30.95	86.6	N/A	3	4
Divert et al., 2015a6	1	2	42	13	30.95	86.6	N/A	3	4
Hussain et al., 2020a1	1	2	100	21	21.00	26.18	21.72	1	2
Hussain et al., 2020a2	1	2	100	21	21.00	26.18	21.72	1	1
Hussain et al., 2020a3	1	2	100	21	21.00	26.18	21.72	1	2
Hussain et al., 2020a4	1	2	100	21	21.00	26.18	21.72	1	1
Hussain et al., 2020a5	1	2	100	21	21.00	26.18	21.72	1	2
Hussain et al., 2020a6	1	2	100	21	21.00	26.18	21.72	1	1
Kaiser et al., 2016a1	1	2	147	73	49.66	23.03	23.03	3	4
Kaiser et al., 2016a2	1	2	147	73	49.66	23.03	23.03	3	4
Kaiser et al., 2016a3	1	2	147	73	49.66	23.03	23.03	3	1
Kaiser et al., 2016a4	1	2	147	73	49.66	23.03	23.03	3	1
Kaiser et al., 2016a5	1	2	147	73	49.66	23.03	23.03	3	4
Kaiser et al., 2016a6	1	2	147	73	49.66	23.03	23.03	3	4
Lock et al., 2016a	1	2	150	41	27.33	35.49	24.81	1	1

Mamalaki et al., 2017a1	1	2	26	26	100	21	23.7	2	4
Mamalaki et al., 2017a2	1	2	26	26	100	21	23.7	2	4
Mamalaki et al., 2017a3	1	2	26	26	100	21	23.7	2	4
Mamalaki et al., 2017a4	1	2	26	26	100	21	23.7	2	4
McCarron et al., 1989	1	2	30	15	50.00	N/A	N/A	1	1
Mekhmoukh et al., 2012a1	1	2	38	38	100	16.5	21.4	1	4
Mekhmoukh et al., 2012a2	1	2	38	38	100	16.5	21.4	1	4
Mekhmoukh et al., 2012b1	1	2	38	38	100	16.4	29.1	1	4
Ragneskog et al., 1996a1	1	2	20	10	50.00	80	N/A	2	4
Ragneskog et al., 1996a2	1	2	20	10	50.00	80	N/A	2	4
Ragneskog et al., 1996a3	1	2	20	10	50.00	80	N/A	2	4
Ragneskog et al., 1996a4	1	2	20	10	50.00	80	N/A	2	4
Ragneskog et al., 1996a5	1	2	20	10	50.00	80	N/A	2	4
Ragneskog et al., 1996a6	1	2	20	10	50.00	80	N/A	2	4
Ragneskog et al., 1996a7	1	2	20	10	50.00	80	N/A	2	1
Ragneskog et al., 1996a8	1	2	20	10	50.00	80	N/A	2	1
Ragneskog et al., 1996a9	1	2	20	10	50.00	80	N/A	2	1
Stroebele et al., 2006a1	1	1	78	15	23.08	20.49	24.87	2	4
Stroebele et al., 2006a2	1	1	78	15	23.08	20.49	24.87	2	4
Stroebele et al., 2006a3	1	1	78	15	23.08	20.49	24.87	2	4
Stroebele et al., 2006a4	1	1	78	15	23.08	20.49	24.87	2	4
Stroebele et al., 2006a5	1	1	78	15	23.08	20.49	24.87	2	4

Supplementary Table S1 (continued)

Study Characteristics

Author (s) and year	Volume	Source of sample	Meal duration	Food type	Experimental setting	Experiment duration	Hedge's *g*	Variance
Divert et al., 2015a1	N/A	3	1	3	2	1	0.29	0.05
Divert et al., 2015a2	N/A	3	1	3	2	1	0.22	0.05
Divert et al., 2015a3	N/A	3	1	1	2	1	-0.34	0.05
Divert et al., 2015a4	N/A	3	1	1	2	1	0.02	0.05
Divert et al., 2015a5	N/A	3	1	1	2	1	0.13	0.05
Divert et al., 2015a6	N/A	3	1	1	2	1	0.39	0.05
Hussain et al., 2020a1	N/A	2	1	1	1	2	-0.29	0.06
Hussain et al., 2020a2	N/A	2	1	1	1	2	-0.33	0.06
Hussain et al., 2020a3	N/A	2	1	1	1	2	0.02	0.06
Hussain et al., 2020a4	N/A	2	1	1	1	2	-0.18	0.06
Hussain et al., 2020a5	N/A	2	1	1	1	2	-0.77	0.06
Hussain et al., 2020a6	N/A	2	1	1	1	2	-0.47	0.06
Kaiser et al., 2016a1	2	2	1	3	1	1	0.28	0.07
Kaiser et al., 2016a2	2	2	1	3	1	1	0.38	0.07
Kaiser et al., 2016a3	2	2	1	3	1	1	0.29	0.07
Kaiser et al., 2016a4	2	2	1	3	1	1	-0.17	0.07
Kaiser et al., 2016a5	2	2	1	3	1	1	0.14	0.05
Kaiser et al., 2016a6	2	2	1	3	1	1	0.32	0.05
Lock et al., 2016a	N/A	4	1	N/A	2	2	0.53	0.03

Mamalaki et al., 2017a1	2	4	2	3	1	2	-0.04	0.08
Mamalaki et al., 2017a2	1	4	2	3	1	2	0.17	0.08
Mamalaki et al., 2017a3	2	4	2	2	1	2	0.07	0.08
Mamalaki et al., 2017a4	1	4	2	2	1	2	0.28	0.08
McCarron et al., 1989	1	4	2	2	1	1	3.08	0.44
Mekhmoukh et al., 2012a1	N/A	1	2	1	1	1	1.78	0.14
Mekhmoukh et al., 2012a2	N/A	1	2	1	1	1	2.08	0.16
Mekhmoukh et al., 2012b1	N/A	1	2	1	1	1	0.34	0.10
Ragneskog et al., 1996a1	2	3	1	1	3	1	0.23	0.09
Ragneskog et al., 1996a2	2	3	1	1	3	1	0.17	0.09
Ragneskog et al., 1996a3	2	3	1	1	3	1	0.19	0.09
Ragneskog et al., 1996a4	2	3	1	1	3	1	0.21	0.09
Ragneskog et al., 1996a5	2	3	1	1	3	1	0.01	0.09
Ragneskog et al., 1996a6	2	3	1	1	3	1	0.65	0.10
Ragneskog et al., 1996a7	1	3	1	1	3	1	0.58	0.10
Ragneskog et al., 1996a8	1	3	1	1	3	1	0.25	0.09
Ragneskog et al., 1996a9	1	3	1	1	3	1	1.23	0.11
Stroebele et al., 2006a1	N/A	2	1	N/A	2	2	0.66	0.03
Stroebele et al., 2006a2	N/A	2	1	N/A	2	2	0.19	0.03
Stroebele et al., 2006a3	N/A	2	1	N/A	2	2	0.35	0.03
Stroebele et al., 2006a4	N/A	2	1	N/A	2	2	0.18	0.03
Stroebele et al., 2006a5	N/A	2	1	N/A	2	2	0.29	0.03

Note. Author (s) and year: a, b, c, d denote that same author(s) published different articles in the same year; a1, a2, b1, b2, b3, and b4 denote that multiple independent samples (or studies) were reported in the same article; Publication type: 1 = journal, 2 = dissertation; Country: 1 = America, 2 = Europe; Experimental design: 1 = between group, 2 = within group, 3 = mixed; Music type: 1 = pop, 2 = classical, 3 = rock, 4 = other types; Volume: 1 = high, 2 = low; Source of sample: 1 = high

school, 2 = university, 3 = nursing home, 4 = other source; Meal duration: 1 = autonomous, 2 = controlled; Food type: 1 = solid, 2 = liquid, 3 = mixed; Experimental setting: 1 = lab, 2 = natural environment, 3 = other; Experiment duration: 1 = long (⩾28 days), 2 = short (< 28 days); N/A = not available.

Supplementary Table S2

Quality Assessment of the Studies Examined

Study	Item 1	Item 2	Item 3	Item 4	Item 5	Item 6	Total	Quality
Divert et al., 2015	6	1	1	6	1	4	19	52.78%
Hussain et al., 2020	6	6	4	6	1	6	29	80.56%
Kaiser et al., 2016	6	6	6	6	6	6	36	100.00%
Lock et al., 2016	6	2	2	1	1	6	18	50.00%
Mamalaki et al., 2017	6	6	5	6	6	6	35	97.22%
McCarron et al., 1989	1	6	4	6	1	5	23	63.89%
Mekhmoukh et al., 2012	6	6	1	6	6	6	31	86.11%
Ragneskog et al., 1996	6	6	5	6	1	6	30	83.33%
Stroebele et al., 2006	2	3	4	4	1	5	19	52.78%

Note:
Item 1 = Was participant eligibility criteria described?
Item 2 = Were the participants randomly selected (or for experimental studies, was the process of randomization clearly described and adequately carried out?);
Item 3 = Were features of music reported?
Item 4 = Were features of food (intake) reported?
Item 5 = Was a power calculation reported and was the study adequately powered to detect hypothesized relationships?
Item 6 = Were critical statistics reported which could be used to calculate effect size?

Supplementary References

References for the Nine Studies Included in the Meta-analysis

Divert, C., Laghmaoui, R., Crema, C., Issanchou, S., Wymelbeke, V. V., & Sulmont-Rossé, C. (2015). Improving meal context in nursing homes. Impact of four strategies on food intake and meal pleasure. *Appetite*, *84*, 139–147. https://doi.org/10.1016/j.appet.2014.09.027

Hussain, M., Egan, H., Keyte, R., & Mantzios, M. (2020). Exploring the Environmental Manifestation of Types of Music on Reinforcing Mindfulness and Concurrent Calorie Intake. *Psychological Reports*, 003329412096727. https://doi.org/10.1177/0033294120967276

Kaiser, D., Silberberger, S., Hilzendegen, C., & Stroebele-Benschop, N. (2016). The influence of music type and transmission mode on food intake and meal duration: An experimental study. *Psychology of Music*, *44*(6), 1419–1430. https://doi.org/10.1177/0305735616636207

Lock, C., Brindal, E., Hendrie, G. A., & Cox, D. N. (2016). Contextual and environmental influences on reported dietary energy intake at evening eating occasions. *Eating Behaviors*, *21*, 155–160. https://doi.org/10.1016/j.eatbeh.2016.03.012

Mamalaki, E., Zachari, K., Karfopoulou, E., Zervas, E., & Yannakoulia, M. (2017). *Presence of music while eating: Effects on energy intake, eating rate and appetite sensations*. 3.

McCarron, A., & Tierney, K. J. (1989). The effect of auditory stimulation on the consumption of soft drinks. *Appetite*, *13*(2), 155–159. https://doi.org/10.1016/0195-6663(89)90112-8

Mekhmoukh, A., Chapelot, D., & Bellisle, F. (2012). Influence of environmental factors on meal intake in overweight and normal-weight male adolescents. A laboratory study. *Appetite*, *59*(1), 90–95. https://doi.org/10.1016/j.appet.2012.03.021

Ragneskog, H., Bråne, G., Karlsson, I., & Kihlgren, M. (1996). Influence of Dinner Music on Food Intake and Symptoms Common in Dementia. *Scandinavian Journal of Caring Sciences*, *10*(1), 11–17. https://doi.org/10.1111/j.1471-6712.1996.tb00304.x

Stroebele, N., & de Castro, J. M. (2006). Listening to music while eating is related to increases in people's food intake and meal duration. *Appetite*, *47*(3), 285–289. https://doi.org/10.1016/j.appet.2006.04.001
